# Evolutionary stasis of the pseudoautosomal boundary in strepsirrhine primates

**DOI:** 10.7554/eLife.63650

**Published:** 2020-11-18

**Authors:** Rylan Shearn, Alison E Wright, Sylvain Mousset, Corinne Régis, Simon Penel, Jean-François Lemaitre, Guillaume Douay, Brigitte Crouau-Roy, Emilie Lecompte, Gabriel AB Marais

**Affiliations:** 1 Laboratoire Biométrie et Biologie Evolutive, CNRS / Univ. Lyon 1VilleurbanneFrance; 2 Department of Animal and Plant Sciences, University of SheffieldSheffieldUnited Kingdom; 3 Faculty of Mathematics, University of ViennaViennaAustria; 4 Zoo de LyonLyonFrance; 5 Laboratoire Evolution et Diversité Biologique, CNRS / Univ. ToulouseToulouseFrance; 6 LEAF-Linking Landscape, Environment, Agriculture and Food Dept, Instituto Superior de Agronomia, Universidade de LisboaLisbonPortugal; Institut Jacques Monod - CNRS UMR7592 - Université Paris Diderot; University of MichiganUnited States

**Keywords:** sex chromosomes, recombination suppression, sexual dimorphism, sexually antagonistic mutations, primates, lemurs, Other

## Abstract

Sex chromosomes are typically comprised of a non-recombining region and a recombining pseudoautosomal region. Accurately quantifying the relative size of these regions is critical for sex-chromosome biology both from a functional and evolutionary perspective. The evolution of the pseudoautosomal boundary (PAB) is well documented in haplorrhines (apes and monkeys) but not in strepsirrhines (lemurs and lorises). Here, we studied the PAB of seven species representing the main strepsirrhine lineages by sequencing a male and a female genome in each species and using sex differences in coverage to identify the PAB. We found that during primate evolution, the PAB has remained unchanged in strepsirrhines whereas several recombination suppression events moved the PAB and shortened the pseudoautosomal region in haplorrhines. Strepsirrhines are well known to have much lower sexual dimorphism than haplorrhines. We suggest that mutations with antagonistic effects between males and females have driven recombination suppression and PAB evolution in haplorrhines

## Introduction

The human sex chromosomes are strongly heteromorphic as they exhibit extensive differences in size, gene number, DNA repeat abundance and heterochromatin composition (Skaletsky et al., 2003; Ross et al., 2005). The X chromosome comprises a large X-specific region recombining only in females whereas the Y comprises a male-specific region that does not recombine at all. Both sex chromosomes share two pseudoautosomal regions (PAR1 and 2) that recombine in both males and females. These sex chromosomes originated from a pair of identical autosomes approximately 150 million years ago, prior to the divergence of placentals and marsupials, with the evolution of Sry – the master male-determining gene in therian mammals – from Sox3 (Lahn and Page, 1999; Skaletsky et al., 2003; Hughes and Rozen, 2012). Since then, at several moments throughout evolutionary history, vast regions of the Y chromosome have stopped recombining with the X, likely through inversions on the Y (Lahn and Page, 1999; Van Laere et al., 2008; Lemaitre et al., 2009; Pandey et al., 2013). These regions show different levels of X-Y divergence and are called evolutionary strata (Lahn and Page, 1999). Strata 1 and 2 are shared among all therians, and stratum three is shared among all placentals (Lahn and Page, 1999; Cortez et al., 2014). The most recent strata (4 and 5) have originated in the history of Catarrhini (Old World monkeys and apes) respectively,~40 and~25 Mya, and now only a very small PAR continues to recombine between X and Y in those primates (Hughes et al., 2012). In humans, PAR1 is the consequence of that process, while PAR2 is a recent addition (Skaletsky et al., 2003).

The process of recombination suppression between sex chromosomes, leading to a reduction in the size of the PAR and formation of evolutionary strata, has been documented in several animal and plant lineages (e.g Nicolas et al., 2005; Zhou et al., 2014; White et al., 2015). Why such a process occurred, however, is unclear. It has been proposed that sexually antagonistic mutations may have favoured the suppression of recombination (Bull, 1983; Rice, 1987; Charlesworth et al., 2005). Theoretical models suggest that if there are male-beneficial/female-detrimental mutations in the PAR, there will be selection to halt recombination, through for example an inversion, to genetically link those mutations to the Y chromosome. Some evidence supporting this hypothesis has recently been found in guppies (Wright et al., 2017), but evidence from a wide range of groups, including primates, is lacking. Furthermore, there are alternative theories for why recombination is halted (reviewed in Charlesworth, 2017; Ponnikas et al., 2018) and so the relative importance of sexual antagonism in sex-chromosome evolution remains unclear.

While previous work on primate sex chromosomes has focused on Haplorrhini (apes, Old and New World monkeys), we studied representatives of the other main primate lineage, the Strepsirrhini (lemurs and lorises). In strepsirrhines, female social dominance (FSD), in which females dominate males, is widespread and likely ancestral (Kappeler and Fichtel, 2015; Petty and Drea, 2015). FSD is associated with increased testosterone production in females, resulting in the masculinization of females, including aspects of their social behaviour and genitalia (Kappeler and Fichtel, 2015; Petty and Drea, 2015). Some species also have rather egalitarian social systems (Pereira and Kappeler, 1997). In addition, sexual size dimorphism is virtually absent among strepsirrhines (Kappeler and Fichtel, 2015; Petty and Drea, 2015). This is in sharp contrast with haplorrhines, where sexual dimorphism is much more pronounced and male-biased; a phenotype that is probably ancient in this group (e.g. Lindenfors, 2002; Kappeler and van Schaik, 2004; Plavcan, 2004). We therefore hypothesized that if male–female differentiation and sexually antagonistic mutations were associated with the degree of X-Y recombination suppression, strepsirrhines should show evidence of less recombination suppression compared to haplorrhines. However, to date, very little is known about the sex chromosomes of strepsirrhines, except that strata 4 and 5 are missing in grey mouse lemurs (*Microcebus murinus*, see Glaser et al., 1999) preventing previous tests of this hypothesis.

To identify the PAB of strepsirrhines, we used an approach relying on sequencing a male and a female at low/moderate depth, mapping the reads to a reference genome and computing the male:female depth ratio (Vicoso and Bachtrog, 2011; Vicoso et al., 2013a; Vicoso et al., 2013b; Zhou et al., 2014). For autosomes, a M:F depth ratio of 1 is expected as males and females have the same copy number of autosomes. On the X chromosome, a ratio of 1 should indicate the PAR that is shared among sexes, a ratio of 0.5 should indicate the X-specific region as males have only one such region and females two, and the boundary between both would indicate the PAB. Using Illumina short-read sequencing technology, we sequenced a male and a female genome in seven species covering the main strepsirrhine lineages representing 65 My of evolution (Pozzi et al., 2014): four Lemuriformes (*Daubentonia madagascariensis* - aye-ayes, *M. murinus* – grey mouse lemur, *Eulemur rubriventer* – red-bellied lemur, *Prolemur simus* – greater bamboo lemur) and three Lorisiformes (*Otolemur garnettii* – northern greater galago, *Galago senegalensis* – senegal bushbaby, *Nyctibebus coucang* – slow loris). The sequencing depth of each sample was between 11.8X and 39.1X (assuming a genome size identical to the human genome) with 78% of the samples being between 20X and 40X, that is moderate sequencing depth (Supplementary file 1A). We then mapped the reads onto publicly available reference genomes of two strepsirrhines (using the human X to scaffold the strepsirrhine X chromosomes) and computed a normalised M:F depth ratio to identify the X-specific region and the PAR on the X chromosome (see Materials and methods).

## Results

**Figure 1.**
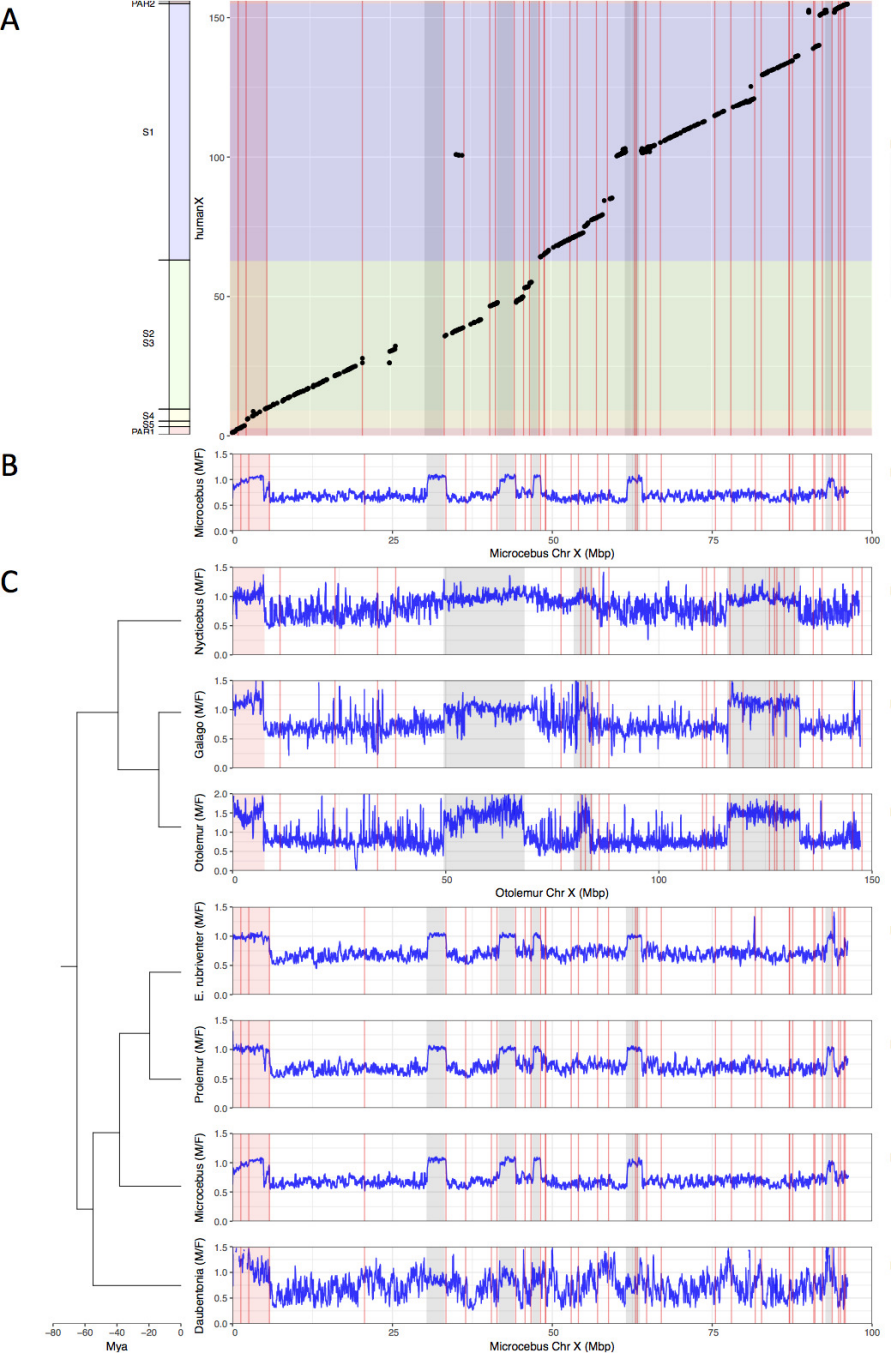
Identification the PAB in seven strepsirrhine species. (**A**) Synteny plot of the human and grey mouse lemur X chromosomes. The human X was used to order the grey mouse lemur scaffolds (see Materials and methods). Black dots represent orthologous genes between the human and grey mouse lemur X chromosomes. Human strata number and boundaries follow Skaletsky et al., 2003 and Hughes and Rozen, 2012. Note that old strata have been split into smaller strata in Pandey et al., 2013. Human strata are indicated by different colours. S4 and S5 are in yellow. PARs are in red.(**B**) M:F read depth ratio along the grey mouse lemur X chromosome. Inferred PAR is shown in red. Regions of elevated M:F coverage ratio (inferred PAR plus other regions in grey) are indicated on panel A plot. (**C**) M:F read depth ratio for all seven strepsirrhine species. Inferred PARs for both the lemurs and the lorises are shown in red. Details on the PAR and the grey regions of the lorises can be found in Figure 1—figure supplement 1. In all panels, red lines indicate scaffold boundaries. See Materials and methods and Supplementary file 1B for the detailed analysis of the regions with elevated M:F coverage ratio shown in grey.

Figure 1A–B shows the results for the grey mouse lemur. Using the human X chromosome to order the grey mouse lemur X scaffolds, we found that the scaffolds corresponding to human PAR1 and strata 4 and 5 have a M:F depth ratio around 1 (Figure 1B), indicating that these regions have remained pseudoautosomal in grey mouse lemurs in agreement with older cytogenetic data (Glaser et al., 1999). The rest of the grey mouse lemur X is X-specific with a M:F ratio close to 0.5. However, five regions in the X-specific region show an elevated ratio. Detailed analysis of these five regions showed that they are fragments of autosomes (see Materials and methods and Supplementary file 1B). It is not clear, however, whether this comes from contamination of the assembly of the X chromosome by autosomal scaffolds or if this has resulted from fusion of autosomal DNA fragments to the PAR during evolution, which are misplaced in the current assembly of the X chromosome. With the fragmented assembly that is available our approach can only reliably identify the PAB, not the size of the PAR. If some autosomal material were translocated to the PAR, and thus enlarging it, it would not be possible to detect it with our approach. Only an improved assembly of the X chromosome in the grey mouse lemur could confirm one of these alternatives. Despite these limitations, it is nonetheless clear that the regions homologous to human PAR1 and strata 4 and 5 are still recombining in grey mouse lemur.

**Figure 1—figure supplement 1.**
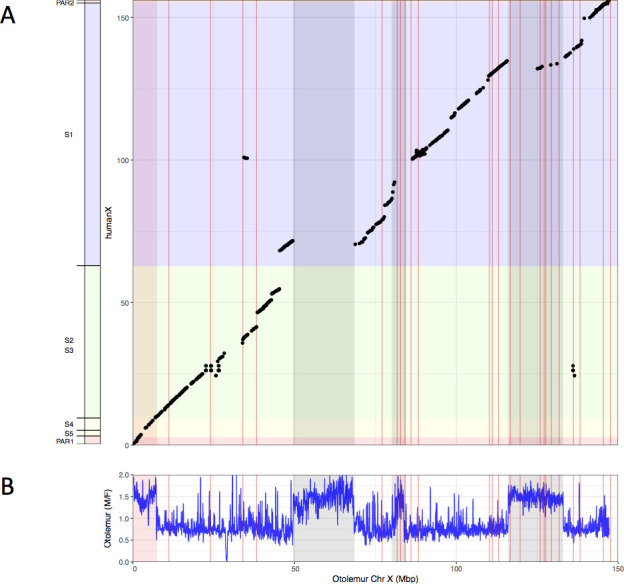
Synteny analysis of northern greater galago and human X chromosomes. (**A**) Synteny plot of the human and northern greater galago X chromosomes. (**B**) M:F read depth ratio along the northern greater galago X chromosome. See legend of Figure 1 for more details.

**Figure 1—figure supplement 2.**
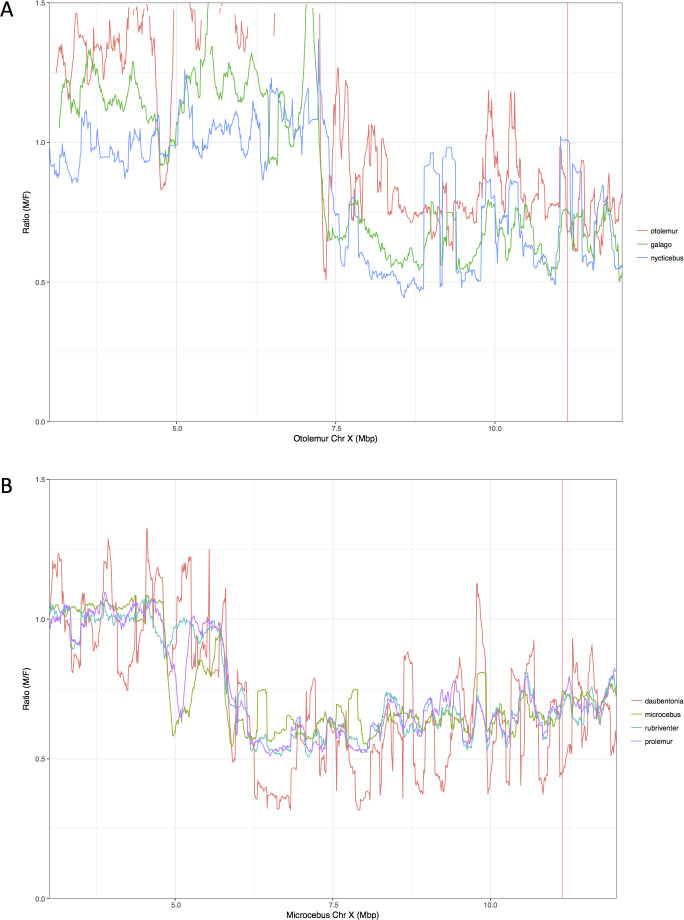
Zoom-in on the inferred PABs. (**A**) Combined M:F read depth ratio for northern greater galago (red), senegal bushbaby (green), slow loris (blue). (**B**) Combined M:F M:F read depth ratio for aye-ayes (red), grey mouse lemur (blue), red-bellied lemur (light blue), greater bamboo lemur (purple). Position of the PABs in lemurs and lorises is the same (see legend of Figure 1 for more details). Positions of the PABs in Mb shown here differ because of differences in the X chromosome assembly between *M. murinus* and *O. garnetti*.

**Figure 2.**
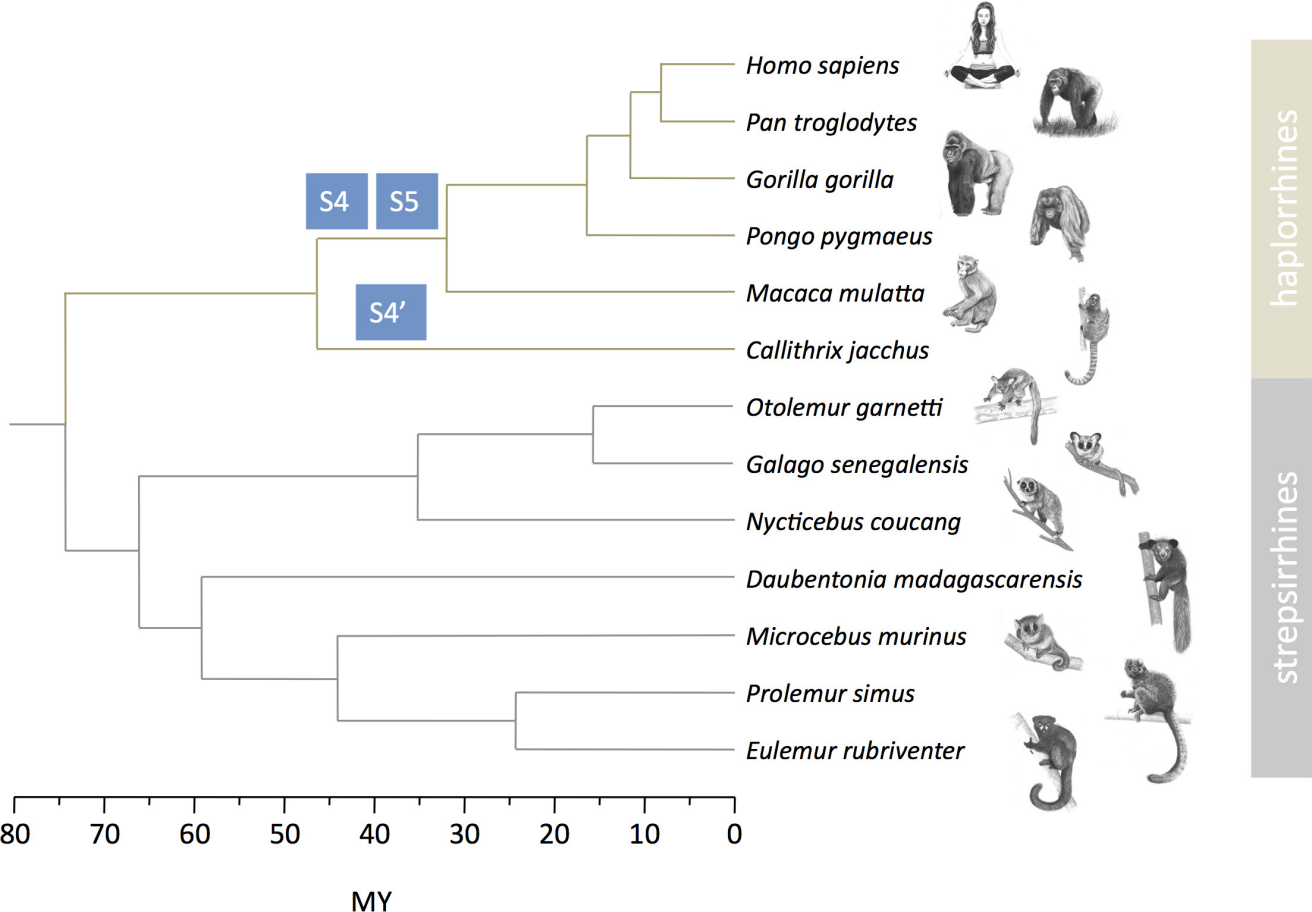
Strata formation in primates. Data on strata in haplorrhines are from Lahn and Page, 1999, Skaletsky et al., 2003, Ross et al., 2005, Hughes and Rozen, 2012, Hughes et al., 2012, Cortez et al., 2014. Data on strepsirrhines are from this study. The phylogenetic tree and divergence times are from Horvath et al., 2008, Pozzi et al., 2014. Drawings of primates were prepared by Philippe Faivre.

We repeated the same analysis for the other six species (Figure 1C). For the lemurs, we used the grey mouse lemur reference genome for the mapping because it is the only one available, and for the lorises, we used the northern greater galago reference genome for the same reason (see Maerials and methods and Figure 1—figure supplement 1 for the dot plot with the human X). Some species are quite distantly related to focal species with the reference genome and so mapping was consequently more difficult. This explains why in some cases the M:F depth ratio is more variable. The results of the aye-ayes analyses are especially noisy because of the large phylogenetic distance to the grey mouse lemur (Figure 1C). However, in all seven species studied here, the pattern is very similar (Figure 1C and a zoom on the PABs in Figure 1—figure supplement 2). All studied strepsirrhines harbour a large pseudoautosomal region including the genes that are in PAR1 and strata 4 and 5 in humans (compare Figure 1A and C for lemurs and Figure 1—figure supplement 1 and C for lorises; both lemur and loris PABs correspond to the boundary between human strata 4 and 3). We can therefore conclude that no suppression of recombination between the X and the Y has occurred in strepsirrhines since the origin of the group >65 millions years ago (Figure 2).

**Figure 3.**
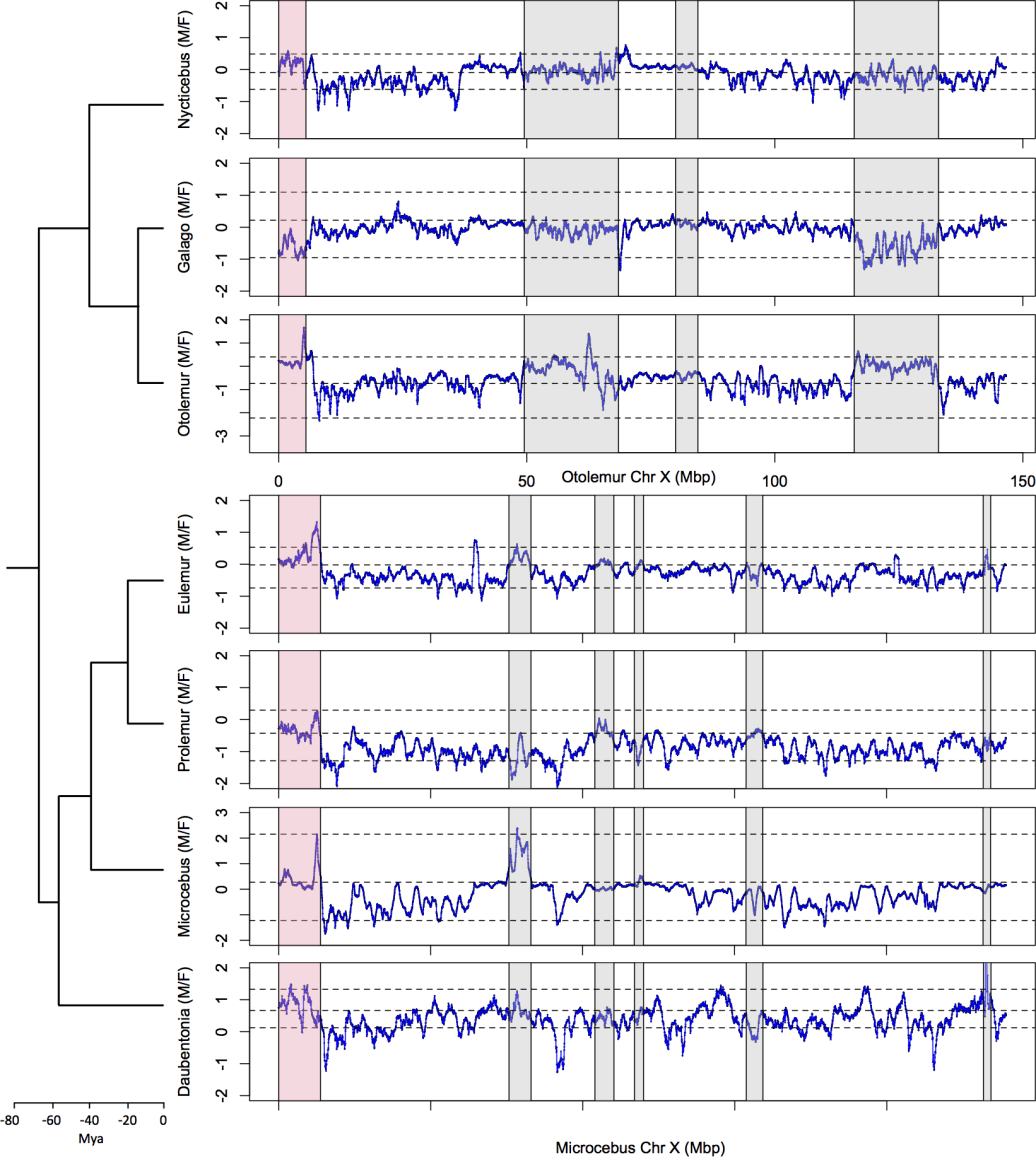
SNP density analysis. M:F SNP density ratio (ln scale) for all seven strepsirrhine species (see Materials and methods for details). Dashed lines indicate the mean M:F SNP density across sliding windows of the same size on chromosome 4, the 97.5 and 2.5% quantiles, to show the variation across the autosomes. See legend of Figure 1 for more details.

It is possible that the M:F read depth approach missed recently evolved strata in strepsirrhines. Recent strata are indeed more difficult to detect with the M:F read depth approach as sex-chromosome divergence can be so low that both X and Y reads map onto the X chromosome and the ratio is close to 1 (Wright et al., 2017). To identify recent strata, we computed the male:female SNP density ratio, which is expected to more effectively detect the PAB when recent strata are present (Vicoso et al., 2013a; Wright et al., 2017). The M:F SNP density ratio is predicted to be one for the PAR, <1 for old strata due to haploidy in males and >1 for recent strata due to accumulation of fixed X-Y differences (Wright et al., 2017). However, our analyses revealed no recent strata in the seven strepsirrhine species studied here (Figure 3).

Our findings are consistent with the hypothesis that recombination suppression between X and Y chromosomes was driven by sexually antagonistic mutations. However, the rate of strata formation is generally low: in primates two strata (4 and 5) were formed in apes and Old World monkeys, one was formed independently in New World monkeys (4’) based on the species studied so far (Hughes et al., 2012; Cortez et al., 2014, and see Figure 2) and our additional data found no new strata formation in strepsirrhines. This observation is consistent with our hypothesis, but could have happened by chance because of a low common rate of strata formation in both suborders. We designed a statistical test to compare the rates of strata formation (expressed in event per My) taking into account the respective divergence times in the haplorrhine and strepsirrhine parts of the phylogenetic tree of the studied species, but this test was only marginally significant (binomial test, p=0.051 see Materials and methods). Because haplorrhines and strepsirrhines have different generation times, comparing rates on a generation-based timescale might however be more relevant. Rescaling time in generations to compare rates of strata formation (expressed in event per million generations) lead to a significantly higher rate in haplorrhines (binomial test, p=0.01 see Materials and methods), consistent with our hypothesis.

We collected phenotypic data from the literature for our set of 13 primate species and confirmed that our sets of strepsirrhine and haplorrhine species differ significantly in sexual dimorphism (teeth and body size, assuming that they reflect the global level of sexual dimorphism in an organism; see Materials and methods and Table 1) but not in sperm competition (testes size, see Materials and methods and Table 1).

**Table 1.** Measures of sexual dimorphism and other features in the set of studied haplorrhine and strepsirrhine species.

Species	Male canine height in mm (sexual selection)	Female canine height in mm (sexual selection)	Refs*	Combined testes mass in g (sperm competition)	Male body mass in g (sperm competition)	Refs*	Male body mass in g (sexual selection)	Female body mass in g (sexual selection)	Refs*	Social and mating system	Refs*
*Callithrix jacchus*	5.08	4.81	[1]	1.3	320	[1]	317	324	[1]	Multimale	[1]
*D. madagascariensis*	NA	NA	NA	NA	NA	NA	2621	2446	[8]	Multimale	[9]
*E. rubriventer*	10.49	9.98	[2]	1.76	2512	[1]	1980	1940	[1]	Monogamous	[1]
*G. senegalensis*	4.01	3.61	[2]	1.66	210	[3]	227	199	[2]	Unimale/Polygynous	[2]
*Gorilla gorilla*	30.26	17.4	[2]	29.6	169000	[1]	170400	71500	[1]	Polygynous	[1]
*Homo sapiens*	10.85	9.97	[2]	40.5	66825	[1]	72100	62100	[1]	Monogamous/Unimale/ Polygynous	[2]
*Macaca mulatta*	16.97	8.13	[2]	46	9200	[4]	11000	8800	[1]	Multimale	[1]
*M. murinus*	2.07	2.08	[2]	2.49	60	[5]	59	63	[2]	Unimale/Polygynous***	[2]
*N. coucang*	7.05	6.8	[2]	1.2	1058	[6]	679	626	[2]	Unimale/Polygynous	[2]
*O. garnettii*	6.53	6.04	[2]	8.93	320	[7]	794	734	[2]	Unimale/Polygynous	[2]
*Pan troglodytes*	21.72	15.26	[2]	128.9	44670	[1]	59700	45800	[1]	Multimale	[1 , 10]
*Pongo pygmaeus*	27	15.95	[2]	35.3	74640	[1]	78500	35800	[1]	Unimale/Multimale	[2]
P. simus**	5.94	5.91	[2]	NA	NA	NA	2532	2248	[8]	Monogamous	[2]

^*^References are [1] Lüpold et al., 2019, [2] Thorén et al., 2006, [3] Gomendio et al., 2011, [4] Harcourt et al., 1995, [5] Lüpold, 2013, [6] Anderson et al., 1999, [7] Dixson and Anderson, 2004, [8] Taylor and Schwitzer, 2011, [9] Mittermeier et al., 2013, [10] Soulsbury, 2010.**or *Hapalemur griseus or H. alaotrensis* ***or multimale, see Soulsbury, 2010. Male body mass values for sperm competition and sexual selection analyses of the same species may differ because they come from different sources (even when a single reference is mentioned).

## Discussion

Our work shows that, during primate evolution, the PAB has remained unchanged in strepsirrhines while several X-Y recombination suppression events have shortened the PAR in haplorrhines. We interpreted this as a consequence of differences in sexual dimorphism, and therefore sexual conflict, in both groups. However, strepsirrhines and haplorrhines differ in many ways and it is of course possible that other aspect(s) of their biology drove the pattern that we found. Strata formation may be influenced for example by gene flow (Matsumoto et al., 2017) and meiotic drive (Scott and Otto, 2017) as suggested recently. Previous work has shown that the genetic diversity of strepsirrhines is highly variable (e.g. Perry et al., 2012). It is however unknown whether strepsirrhines and haplorrhines exhibit systematic differences in gene flow rates and meiotic drive dynamics. A limit of this work is the use of a qualitative description of sexual dimorphism and not a quantitative one, which we could have compared to the number of strata. Future work could explore strata formation in more species and gain sufficient statistical power to compare the number of strata to phenotypic data on sexual dimorphism in primates using trait-evolution phylogenetic methods, which requires large datasets.

Evidence for the sexually antagonistic mutations hypothesis has been found in other organisms. In guppies, while the Y chromosome exhibits low levels of divergence from the X (Wright et al., 2017; Bergero et al., 2019; Darolti et al., 2019), populations exhibiting stronger sexual dimorphism seem to have a larger non-recombining region (Wright et al., 2017; Wright et al., 2019; Almeida et al., 2020). In the brown alga *Ectocarpus*, sexual dimorphism is extremely low and as expected sex chromosomes are homomorphic, with a small non-recombining region, despite being very old (Ahmed et al., 2014). It should be noted, however, that other forces might be driving the process of strata formation in some lineages. In ruminants, the PAR seems to have undergone a process of attrition due to accumulation of DNA repeats (Van Laere et al., 2008; Raudsepp and Chowdhary, 2016). In *Microbotryum violaceum*, strata are found on the mating-type chromosomes despite the fact that this species only has mating types and not sexes, such that sexual antagonism is absent (Branco et al., 2017). Thus, sexually antagonistic mutation may not be a ubiquitous explanation of strata formation in all organisms.

Although sexual dimorphism is generally low in strepsirrhines, there are some differences among species in this lineage, with the genus *Eulemur* exhibiting the most pronounced sexual dimorphism (Petty and Drea, 2015). In these species, including the red-bellied lemur (*E. rubriventer*), which was analysed here, males and females exhibit striking sexual dichromatism, that is they differ in pelage colouration (Rakotonirina et al., 2017). The red-bellied lemur did not show more evidence for recombination suppression than the other species studied here. Sexual dichromatism may rely on sexually antagonistic mutations. The antagonism might have been solved not through Y-linkage but instead through sex-biased expression for example (Ellegren and Parsch, 2007; Gazda et al., 2020). Future research could focus on sex-biased expression in strepsirrhines to test these ideas.

## Materials and methods

### Research plan

To test whether recombination suppression is less frequent on strepsirrhine sex chromosomes compared to haplorrhines, we selected strepsirrhine species that would maximise the representation of this group’s diversity, and that were also readily accessible. We then sequenced a male and female of each species and mapped the obtained male and female reads to a reference X chromosome. The male to female depth ratio was then computed along the length of the X chromosome and the PAB was identified as the boundary between zones with a ratio of one (indicative of the PAR) and zones with a ratio of 0.5 (indicative of the non-recombining region).

### Sampling

We selected seven species covering as much phylogenetic diversity of Strepsirrhini as possible (see Supplementary file 1A). Both infra-orders (Lemuriformes and Lorisiformes) are equally represented. A male and a female individual were sampled for all species (except *O. garnettii*, the northern greater galago, for which sequence data from a female individual were retrieved from NCBI, see Supplementary file 1A). Blood samples of *E. rubriventer* (red-bellied lemur) and *P. simus* (greater bamboo lemur) were collected from living animals at Zoo de Lyon in EDTA blood collection tubes to avoid coagulation. Hair samples (with follicles and roots) of the female *Daubentonia madagascarensis* (aye-aye) were collected from a living animal at Zoo Frankfurt. Samples of *M. murinus* belong to the Brunoy laboratory (UMR7179, France; agreement E91-114-1 from the Direction Départementale de la Protection des Populations de l’Essonne): the biopsies were obtained from muscle tissues after the animals’ natural death. Tissues samples of a male *D. madagascariensis*, and samples of *G. senegalensis* (Senegal bushbaby), *Nycticebus coucang* (slow loris) and of a male *O. garnettii* were obtained from the tissues and cryopreserved cell collection of the National Museum of Natural History (MNHN, Paris, see Supplementary file 1A).

### DNA extraction and sequencing

DNA from *E. rubriventer*, *P. simus* and female *D. madagascariensis* were extracted using two different Macherey Nagel kits. Blood samples were treated with NucleoSpin Blood Quickpure kit. Hair samples were treated with NucleoSpin DNA trace kit after a mechanical crushing of hair bulbs. DNA from the tissues and cells samples (for other species) was extracted using the DNeasy Blood and Tissue kit (Qiagen) following the manufacturer’s instructions. DNA was stored at −20° C and sent on dry ice to the sequencing platform.

A genomic DNA library was constructed for each sample using Illumina kits (TruSeq nano LT for Hiseq 2500 and 3000 sequencing). Paired-end sequencing was conducted using an Illumina Hiseq 2500 (2 × 125 bp) or 3000 (2 × 150 bp) with one or two individuals per lane at Genotoul, the INRA sequencing platform in Toulouse. Sequences were all found to be of high quality (using FastQC, https://www.bioinformatics.babraham.ac.uk/projects/fastqc) and without contamination. Consequently, no trimming was done. Sequence data and coverage are shown in Supplementary file 1A.

### Chromosome assembly

Reference X chromosomes were not available for any species and genome assemblies were only available for two species that were (1) closely related to, or the same as the species being studied, and (2) assembled to an extent that it would be possible to construct a de novo X chromosome. These were *M. murinus* (grey mouse lemur, Mmur_2.0 version from NCBI) and *O. garnettii* (northern greater galago, OtoGar4 version from NCBI).

De novo X chromosomes were constructed for these species using scaffolds from whole genome assemblies on NCBI, which were selected, ordered and oriented against the human X chromosome. This was achieved using SynMap, an online software pipeline within the CoGe toolkit (Lyons and Freeling, 2008; Lyons et al., 2008) that identified putative homologous genes between potential X scaffolds and the human X chromosome with a blast comparison (Altschul et al., 1990) using the Last algorithm (a variant of Blastz, see Schwartz et al., 2003). An algorithm within the SynMap pipeline then identified a colinear series of homologous genes between potential X scaffolds and the human X chromosome as regions of synteny, and these were arranged in order accordingly. The relative gene order DAGChainer option was used, with a maximum distance of 20 genes between two matches and a minimum of five aligned pairs of genes. The human X chromosome reference was sourced from the GRCh37.p13 Primary Assembly on NCBI (Reference Sequence: NC_000023.10).

As the results of some of the analyses in this study required normalisation using an autosome from the corresponding species, a reference autosome was constructed using the same process. In this case, the human chromosome four was used to construct a de novo chromosome four for *M. murinus* and *O. garnettii*, which was selected for its similar size to the X chromosome.

### Read mapping

Male and female reads for each species were aligned separately to their most closely related de novo X chromosome using Bowtie version 2–2.2.7 (Langmead et al., 2009). The reads were then sorted according to their position on the de novo X chromosome using Samtools version 1.3.1 (Li et al., 2009; Li, 2011).

### Coverage analysis

Read depth was calculated for each sex at each position from the mapped reads on the de novo X using Samtools. The coverage for each sex was then normalised by dividing the depth at each position by the mean coverage depth for that species and sex on an autosome (chromosome four). The ratio of normalised male to female coverage was then calculated at each position and the data was summarised as a sliding window average using a window size of 150 kb sliding at increments of 10 kb or larger windows and increments depending on the species. This data manipulation was performed using AWK version 4.1.3.

### Analysis of the regions of the strepsirrhine X chromosomes with unusual male:female coverage ratio

In Figure 1, both lemur and loris X chromosomes exhibit regions with male:female coverage ratio close to 1 (shown in grey) in their X-specific parts, where a ratio of 0.5 is expected. The grey mouse lemur has five such regions, the northern greater galago three. The dot plots of the strepsirrhine and the human X chromosomes (see Figure 1 and Figure 1—figure supplement 1) clearly show that little or no homologous genes are found in those regions, which suggest that they may be homologous to other human chromosomes. This would be consistent with the male:female coverage ratio of 1, typical of autosomal regions, that we found for these regions. To explore this possibility, we extracted the sequences of those regions and performed a tblastn against all the human proteins (human genome version GRCh38). In case of isoforms, the longest protein was kept so that a human gene was present only once. We then filtered the tblastn results by keeping only hits with >80% similarity (based on average nucleotide divergence between lemurs and humans) and e-value <10^–9^. From those, we kept human proteins covered by hits to >80% using SiLix (Miele et al., 2011). Only proteins matching to no more than one region were kept. The results of the tblastn are shown in the supplementary file 1B.

For all regions except one, most homologs that we identified are from the human autosomes, which confirms our hypothesis. These homologs are mainly a few sources: chromosomes 1, 8 and 12 for regions 46.8–48, 61.5–63.7, 92.7–93.7 and 41.6–44.1 of the grey mouse lemur X chromosome, and chromosomes 12, 13 and 20 for regions 80–84.5, 116–133 and 49.5–68.5 of the northern greater galago X chromosome. These results can be interpreted two ways. One possibility is that the assemblies of the lemur and loris X chromosomes wrongly include autosomal scaffolds. Another possibility is that during the evolution of strepirrhines, some autosomal fragments have been translocated to the PAR, and the assembly failed to order these fragments correctly. Our approach cannot tell apart these possibilities but in all cases, our results suggest that these regions are probably assembly errors.

Changing tblastn outputs filtering did not change qualitatively the results. With lower %identity thresholds, we detected autosomal homologs for region 30.3–33.2 (for example, with %identity >65, we found two proteins from chrom. 1, one from chrom. 2 and 1 from chrom 19).

### SNP density analysis

To detect potential regions that may have stopped recombining between strepsirrhine X and Y chromosomes relatively recently, the difference in male to female SNP density was examined for all species. For each sex of each species, SNPs were called from the mapped reads using Samtools mpileup and then converted to profiles using sam2pro version 0.8 from the mlRho package (Haubold et al., 2010). Specifically, sites with coverage <5 were excluded from the analysis and SNPs were called when a site had a minor allele frequency of 0.3 times the site coverage. The ratio of male to female SNP density was calculated for 600 kb sliding windows at increments of 10 kb. 0.001 was added to allow for a Log transformation and male to female SNP density was calculated at each window as Log(sum male SNPs) – Log(sum female SNPs). This calculation was performed using R version 3.3.2. We also calculated SNP density across an autosome (chromosome four) using the same approach and computed mean male to female SNP density and 97.5% and 2.5% quantiles across all windows.

### Statistical test on strata formation

We partitioned the phylogenetic tree with total branch length *Δt* into two subtrees with branch lengths *Δt_1_* and *Δt_2_*, *Δt = Δt_1_+Δt_2_*. Assuming a constant rate *λ* for the formation of new evolutionary stratum, the number *S* of new strata formed during a time interval *Δt* is Poisson-distributed with parameter *λΔt*

(1) $$P(S=k)=\frac{(\lambda \Delta t{)}^{k}e^{-\lambda \Delta t}}{k!}.$$

On the subtree *i*, during the time interval *Δt_i_* we observe the formation of *S_i_* new strata. We wanted to contrast the following two hypotheses:

- *H_0_*: Strata accumulated at a common rate *λ_0_* on both parts of the tree.
- *H_1_*: Strata accumulated at different rates *λ_i_* during the time intervals *Δt_i_*.

We used the number *S_1_* of strata formed in the time interval *Δt_1_* as the test statistics and compute the conditional probability to observe a larger value given the total number *S_1_ +S_2_* of strata formed in the time interval *Δt_1_+Δt_2_* under the null hypothesis *H_0_*:

(2) $$\begin{array}{ll} P(S_{1}\geq k_{1}|S_{1}+S_{2}=k_{1}+k_{2},\Delta t_{1},\Delta t_{2}) & =\frac{(k_{1}+k_{2})!}{(\lambda_{0}(\Delta t_{1}+\Delta t_{2}){)}^{k_{1}+k_{2}}e^{-\lambda_{0}(\Delta t_{1}+\Delta t_{2})}}\\ & \times \sum_{j=k_{1}}^{k_{1}+k_{2}}\frac{\lambda_{0}^{K_{1}+k_{2}}\Delta t_{1}^{j}\Delta t_{2}^{k_{1}+k_{2}-j}e^{-\lambda_{0}(\Delta t_{1}+\Delta t_{2})}}{j!(k_{1}+k_{2}-j)!}\\ & =\sum_{j=k_{1}}^{k_{1}+k_{2}}(\begin{array}{c} k_{1}+k_{2}\\ j \end{array})(\frac{\Delta t_{1}}{\Delta t_{1}+\Delta t_{2}}{)}^{j}(\frac{\Delta t_{2}}{\Delta t_{1}+\Delta t_{2}}{)}^{k_{1}+k_{2}-j} \end{array},$$

where we recognized the binomial distribution. Note that this probability is independent of the common rate *λ_0_* of the Poisson process. Applying this test is conceptually equivalent to tossing an unbalanced coin *k_1_+k_2_* times with a probability *p = Δt_1_ / (Δt_1_+Δt_2_*) to get a head and computing the probability to obtain at least *k_1_* times a head.

The phylogenetic relationships and mean divergence times for the included primate species were recovered from a previously published primate phylogeny and divergence dates (Pozzi et al., 2014). Detailed phylogenetic relationships among strepsirrhine lineages (Horvath et al., 2008) were used to infer phylogenetic relationships in the cases when species in our analysis were not included in this reference study. The divergence times are shown in a see Supplementary file 1C. Generation times in the studied primate species are highly variable, and we are mostly interested in comparing *per generation* rather than *per year* rates of strata formation. For this purpose, the branch lengths in the phylogenetic tree needed to be rescaled by the generation times. We used the age at first reproduction as a proxy for generation time following the example of Gaillard et al., 2005. Ages at first reproduction for the extant species in the phylogenetic trees were obtained from Ernest, 2003 and maximum-likelihood estimates of this trait were obtained for internal nodes of the phylogenetic tree with the fastAnc method implemented in phytools (Revell, 2012). This method assumes that the age at first reproduction evolves neutrally according to a Brownian motion model (Felsenstein, 1973; Schluter et al., 1997). The branch lengths of the phylogenetic tree were rescaled by the generation times. In order to take into account variable generation time along a branch, we used the following method: We denoted *g* the time counted in generations and *t* the time counted in years along a phylogenetic branch. The instantaneous generation time (expressed in years per generation) along a given branch at any time *t* is *γ(t) = dt / dg.* We assume a linear trend for *γ(t)* between an ancestral node (at *t = t_a_*, for which *γ(t_a_) = γ_a_*) and a descendant node (at *t = t_d_*, for which *γ(t_d_) = γ_d_*). This assumption raised the following ordinary differential equation:

$$\gamma (t)=\frac{dt}{dg}=\gamma_{a}+\lambda (t-t_{a}),$$

where *λ* = *(γ_d_ - γ_a_*) / *(t_d_ - t_a_*). The general form for the solutions of this equation is

$$g(t)=\frac{1}{\lambda }\ln(\gamma_{a}+\lambda (t-t_{a}))+K,$$

where *K* is an integration constant. The number of generations elapsed on the branch between times *t_a_* and *t_d_* is thus

(3) $$g(t_{d})-g(t_{a})=\frac{t_{d}-t_{a}}{\gamma_{d}-\gamma_{a}}\ln\frac{\gamma_{d}}{\gamma_{a}}.$$

The ages at first reproduction for the extant species and their maximum-likelihood estimates as well as the rescaled branch lengths in the primate phylogeny are shown in Supplementary file 1D.

The haplorrhine lineages in our sample have evolved for *Δt_1_* = 188.52 My (44.23 million generations) during which *S_1_* = 3 new strata were formed. The strepsirrhine lineages evolved for *Δt_2_* = 321.32 My (158.52 million generations) and no new strata was formed (*S_2_* = 0). Comparing the rates of strata formation expressed in number of events per million year lead to a marginally significant p-value (one-tailed binomial test, p=0.051), this trend became significant when considering the rates expressed in number of events per million generations (one-tailed binomial test, p=0.010).

### Statistical analysis of phenotypic differences among primates

All statistical analyses were conducted with the R statistical software (R Development Core Team, 2019). Sexual dimorphism based on body mass (SSD, size-based sexual dimorphism) or on canine length (CSD, canine height based sexual dimorphism) was quantified as the logarithm of the ratio of the male to the female values (for instance, SSD = ln(male body mass/female body mass), Plavcan, 2004). The relative testes mass (RTM) was computed as the residual of the linear regression ln(combinedtestesmass)∼ln(malebodymass).

In a first approach, the phylogenetic architecture underlying the data was ignored and we simply compared the average dimorphism value between the two groups (haplorrhines vs strepsirrhines). In a second stage, we accounted for the underlying phylogenetic architecture using phylogenetic contrasts in a classical phylogenetic generalised least square analysis (see Symonds and Blomberg, 2014). Two evolutionary models were investigated: a simple Brownian motion (BM) and the Ornstein-Uhlenbeck model (OU) that includes stabilizing selection. The results based on the latter (OU) model should however be considered cautiously as this analysis is certainly over-parameterized considering the very small sample size (between n = 11 and n = 13 species). Analyses accounting for phylogenetic architecture in the data used the following specialized R packages:adephylo (Jombart and Dray, 2010), ape (Paradis and Schliep, 2019), geiger (Harmon et al., 2008) and phytools (Revell, 2012).

Sexual dimorphism based on body mass (SSD, mean ± standard error) was 0.378 ± 0.097 in haplorrhines and 0.062 ± 0.017 in strepsirrhines. This difference based on n = 13 observations was statistically significant only when ignoring phylogenetic inertia (p=0.043) but no longer significant when considering phylogenetic inertia with a Brownian motion model (p=0.66). Analysis involving an OU model would lead to a significant difference between the two groups (p=0.043) but this analysis may either be over-parameterized or suffer from the lack of phylogenetic signal in our data as revealed by the low Pagel’s λ <0.001 (not significantly different from 0) estimated in the Brownian motion model. In such a case, non phylogenetically-corrected analyses should be reported (Freckleton, 2009).

Sexual dimorphism based on canine height (CSD) showed the same kind of pattern: the mean is 0.385 ± 0.076 in haplorrhines and 0.045 ± 0.016 in strepsirrhines. This difference based on n = 12 observations is only significant when ignoring the underlying phylogeny (p=0.013) but no longer significant (p=0.39) when phylogeny is accounted for with a Brownian motion model (leading to a non different from 0 estimate of Pagel’s λ). The OU model leads to a significant difference between groups (p=0.013).

Based on our n = 11 observations, the average relative testes mass did not significantly differ between haplorrhines (0.18 ± 0.24) and strepsirrhines (−0.21 ± 0.32). In order to avoid using residuals of a generalised least square model, we also compared testes mass in an analysis of covariance model (see Lemaître et al., 2009, for an example) including the male body mass as a covariate using the following statistical model in R: ln(combinedtestesmass)∼ln(malebodymass)+group. The results were however qualitatively unchanged (the p-value associated with the ‘group’ factor was p=0.4).

### Data and code accessibility

All the data generated in this study is available at NCBI (project # PRJNA482296). Scripts for the entire coverage analysis pipeline (suitable for compute clusters using Torque job scheduling) are available on GitHub (https://github.com/rylanshearn/sex-read-depth; Shearn, 2018; copy archived at swh:1:rev:0e33f6b8158f4e1385af58117afeb762576cc0fb). 

## Data Availability

All the data generated in this study is available at NCBI (project # PRJNA482296). The following dataset was generated: ShearnR2018Strepsirrhini Raw sequence readsNCBI BioProjectPRJNA482296 The following previously published datasets were used: Broad Institute2006Genome sequencing of Otolemur garnettiiENA ProjectPRJNA16955 Broad Institute2007Microcebus murinus RefSeq GenomeNCBI BioProjectPRJNA285159 Broad Institute2012Otolemur garnettii RefSeq GenomeNCBI BioProjectPRJNA169348 Genome Reference Consortium2013Human genomeNCBIGRCh37.p13

## Additional files

Supplementary file 1. Supplemetary Tables.

Transparent reporting form
