# Supplementary material for: Evolutionary stasis of the pseudoautosomal boundary in strepsirrhine primates
Source: eLife. 2020 Nov 18;9:e63650. doi: 10.7554/eLife.63650 (PMC7717902; doi:10.7554/eLife.63650)
Supplement: Supplementary file 1. [file elife-63650-supp1.docx]

Section A: Statistics about the genome sequencing in the 7 species.

| **Lineage** | **Genus** | **Species** | **Common name** | **Sex** | **Read #** | **Read length** | **Sequences (Gb)** | **Coverage (X)*** | **Source** |
| --- | --- | --- | --- | --- | --- | --- | --- | --- | --- |
| Lemuriformes | *Daubentonia* | *madagascariensis* | aye-aye | M | 574 860 296 | 125 | 71.9 | 23.2 | MNHN, Paris |
| Lemuriformes | *Daubentonia* | *madagascariensis* | aye-aye | F | 807 533 380 | 150 | 121.1 | 39.1 | Zoo Frankfurt |
| Lemuriformes | *Microcebus* | *murinus* | gray mouse lemur | M | 553 217 340 | 125 | 69.2 | 22.3 | MNHN, Brunoy |
| Lemuriformes | *Microcebus* | *murinus* | gray mouse lemur | F | 567 375 076 | 125 | 70.9 | 22.9 | MNHN, Brunoy |
| Lemuriformes | *Eulemur* | *rubriventer* | red-bellied lemur | M | 361 251 832 | 150 | 54.2 | 17.5 | Zoo de Lyon |
| Lemuriformes | *Eulemur* | *rubriventer* | red-bellied lemur | F | 316 639 574 | 150 | 47.5 | 15.3 | Zoo de Lyon |
| Lemuriformes | *Prolemur* | *simus* | greater bamboo lemur | M | 242 884 578 | 150 | 36.4 | 11.8 | Zoo de Lyon |
| Lemuriformes | *Prolemur* | *simus* | greater bamboo lemur | F | 428 087 286 | 150 | 64.2 | 20.7 | Zoo de Lyon |
| Lorisiformes | *Nyctibebus* | *coucang* | slow loris | M | 665 798 842 | 150 | 99.9 | 32.2 | MNHN, Paris |
| Lorisiformes | *Nyctibebus* | *coucang* | slow loris | F | 670 569 564 | 150 | 100.6 | 32.4 | MNHN, Paris |
| Lorisiformes | *Galago* | *senegalensis* | senegal bushbaby | M | 641 724 580 | 150 | 96.3 | 31.1 | MNHN, Paris |
| Lorisiformes | *Galago* | *senegalensis* | senegal bushbaby | F | 666 087 196 | 150 | 99.9 | 32.2 | MNHN, Paris |
| Lorisiformes | *Otolemur* | *garnetti* | northern greater galago | M | 662 474 900 | 150 | 99.4 | 32.1 | MNHN, Paris |
| Lorisiformes | *Otolemur* | *garnetti* | northern greater galago | F | 2 599 993 104 | 100 | 260.0 | 83.9 | EBI** |

*based on human genome size (assuming similar genome sizes in humans and all these species)

**SRR016877 to SRR016896 files fastq.gz 1 and 2.

Section B: results of the blast of the regions with unusual male:female coverage ratio against the human genome

|  | *Microcebus murinus* X chromosome regions* | | | | | *Otolemur garnetti* X chromosome regions* | | |
| --- | --- | --- | --- | --- | --- | --- | --- | --- |
| Human chromosomes | 30.3-33.2 | 41.6-44.1 | 46.8-48 | 61.5-63.7 | 92.7-93.7 | 49.5-68.5 | 80-84.5 | 116-133 |
| Chrom. 1 |  |  | **4** | **54** |  | 13 | 2 | 1 |
| Chrom. 2 |  |  |  | 2 |  | 4 | 1 |  |
| Chrom. 3 |  |  |  |  |  |  | 1 |  |
| Chrom. 4 |  |  |  |  |  | 2 |  |  |
| Chrom. 5 |  |  |  | 2 |  | 2 | 1 |  |
| Chrom. 6 |  |  |  |  |  | 10 |  |  |
| Chrom. 7 |  |  |  |  |  |  |  | 2 |
| Chrom. 8 |  |  | 1 | 1 | **4** | 1 | 1 | 1 |
| Chrom. 9 |  |  |  |  |  |  | 1 |  |
| Chrom. 10 |  |  |  |  |  |  |  |  |
| Chrom. 11 |  |  |  |  |  |  |  |  |
| Chrom. 12 |  | **8** | 1 | 2 |  | 3 | **15** |  |
| Chrom. 13 |  |  |  |  |  | 1 | 1 | **44** |
| Chrom. 14 |  |  |  |  |  | 4 | 1 | 1 |
| Chrom. 15 |  |  |  | 2 |  | 2 |  |  |
| Chrom. 16 |  |  |  | 1 |  | 1 |  | 1 |
| Chrom. 17 |  | 1 |  |  |  | 3 | 1 |  |
| Chrom. 18 |  |  |  |  |  | 2 |  |  |
| Chrom. 19 |  |  |  | 1 |  | 3 |  |  |
| Chrom. 20 |  |  |  |  |  | **119** |  |  |
| Chrom. 21 |  |  |  |  |  |  |  |  |
| Chrom. 22 |  |  |  | 1 |  | 1 |  |  |
| Chrom. X |  |  | 2 | 1 |  | 2 | 3 |  |

*coordinates in Mb

Human chromosome with the largest number of homologs is shown in bold

Section C: Divergence time estimates in the primate phylogeny.

| Node label^a^ | Mean age^a^ (My) | Descendant nodes or species | |
| --- | --- | --- | --- |
| 4 | 7.65 | Homo sapiens | *Pan troglodytes* |
| 5 | 10.63 | 4 | *Gorilla gorilla* |
| 7 | 17.29 | 5 | *Pongo pygmaeus* |
| 38 | 32.12 | 7 | *Macaca mulatta* |
| 44^b^ | 46.72 | 38 | *Callithrix jacchus* |
| 61 | 74.11 | 44 | 60 |
| 50^c^ | 24.24 | *Eulemur rubriventer* | *Prolemur simus* |
| 54^d^ | 43.46 | 50 | *Microcebus murinus* |
| 55 | 59.55 | 54 | *Daubentonia madagascarensis* |
| 56 | 17.28 | *Galago senegalensis* | *Otolemur garnetti* |
| 58 | 36.35 | 56 | *Nycticebus cougang* |
| 60 | 66.33 | 55 | 58 |

^a^ For sake of clarity we report the node labels and ages from our reference primate phylogeny (Pozzi et al. 2014).

^b^ *Callithrix jacchus* is not included in the reference phyolgeny but the divergence time between platyrrhini and catarrhini can be used.

^c^ *Prolemur simus* and *Eulemur rubriventer* are not included in the reference phylogeny, but the divergence between *Eulemur* *macaco* and *Lemur catta* can be used (node 18 in Horvath et al. 2008).

^d^ *Microcebus murinus* is not included in the reference phylogeny, but the divergence with *Lepilemur* sp. can be used (node 7 in Horvath et al. 2008).

Section D: Rescaling of the primate phylogeny with generation time.

| Ancestral node | | Descendant node | | Branch length | |
| --- | --- | --- | --- | --- | --- |
| Node^a^ | AFR^b^ | Node^a^ | AFR^b^ | My | MGen |
| 4 | 119.12 | *H. sapiens* | 131.87 | 7.65 | 0.73 |
| 4 | 119.12 | *P. troglodytes* | 131.87 | 7.65 | 0.73 |
| 5 | 109.18 | 4 | 119.12 | 2.98 | 0.31 |
| 5 | 109.18 | *G. gorilla* | 89.33 | 10.63 | 1.29 |
| 7 | 99.42 | 5 | 109.18 | 6.66 | 0.77 |
| 7 | 99.42 | *P. pygmaeus* | 110.12 | 17.29 | 1.98 |
| 38 | 68.49 | 7 | 99.42 | 14.83 | 2.14 |
| 38 | 68.49 | *M. mulatta* | 41.08 | 32.12 | 7.19 |
| 44 | 50.51 | 38 | 68.49 | 14.6 | 2.97 |
| 44 | 50.51 | *C. jacchus* | 16.33 | 46.72 | 18.52 |
| 61 | 36.80 | 44 | 50.51 | 27.39 | 7.59 |
|  |  |  |  |  |  |
| Total length for the haplorrhine subtree (*Δt_1_*) | | | | 188.52 | 44.23 |
|  |  |  |  |  |  |
| 61 | 36.80 | 60 | 32.91 | 7.78 | 2.68 |
| 60 | 32.91 | 58 | 23.70 | 29.98 | 12.82 |
| 60 | 32.91 | 55 | 31.60 | 6.78 | 2.52 |
| 58 | 23.70 | 56 | 17.99 | 19.07 | 11.05 |
| 58 | 23.70 | *N. cougang* | 23.42 | 36.35 | 18.51 |
| 56 | 17.99 | *O. garnetti* | 19.48 | 17.28 | 11.07 |
| 56 | 17.99 | *G. senegalensis* | 11.33 | 17.28 | 14.40 |
| 55 | 31.60 | *D. madagascarensis* | 39.00 | 59.55 | 20.32 |
| 55 | 31.60 | 54 | 26.49 | 16.09 | 6.67 |
| 54 | 26.49 | 50 | 26.39 | 19.22 | 8.72 |
| 54 | 26.49 | *M. murinus* | 12.90 | 43.46 | 27.61 |
| 50 | 26.39 | *P. simus* | 28.93 | 24.24 | 10.52 |
| 50 | 26.39 | *E. rubriventer* | 23.73 | 24.24 | 11.62 |
|  |  |  |  |  |  |
| Total length for the strepsirrhine subtree (*Δt_2_*) | | | | 321.32 | 158.52 |

^a^ For sake of clarity we report the node labels of our reference primate phylogeny (Pozzi et al. 2014).

^b^ Values of the age at first reproduction (AFR, expressed in months) for extant species are from Ernest (2003). The value for *H. sapiens* was not available and conservatively set to the same value as for *P. troglodytes*. The value for *E. rubriventer* was not available and was estimated as the mean value of other species in the same genus. The values for internal nodes of the primate phylogeny are maximum-likelihood estimates under a Brownian motion model (Felsenstein 1973; Schluter et al. 1997) obtained with phytools (Revell 2012).
