# Supplementary material for: Evolutionary stasis of the pseudoautosomal boundary in strepsirrhine primates
Source: eLife. 2020 Nov 18;9:e63650. doi: 10.7554/eLife.63650 (PMC7717902; doi:10.7554/eLife.63650)
Supplement: Peer review history

# Decision letter

**DOI:** 10.7554/eLife.63650.sa1

**Affiliations:** Institut Jacques Monod - CNRS UMR7592 - Université Paris Diderot

In the interests of transparency, eLife publishes the most substantive revision requests and the accompanying author responses.

[Editors' note: This article has been reviewed and recommended by Peer Community in Evolutionary Biology (https://doi.org/10.24072/pci.evolbiol.100108).]

**Acceptance summary:**

The Y chromosome tends to diverge rapidly in regions that do not recombine with the X chromosome. Sexually antagonistic selection, when sexes exhibit conflicting reproductive optima, has been hypothesized to increase recombination suppression between sex chromosomes. The authors found that the size of the non-recombining region has remained stable in lemurs and lorises, which display low sexual dimorphism, while it has expanded in the ancestors of apes and monkeys, which exhibit high sexual dimorphism. This elegant work provides empirical support for sexual antagonism shaping sex chromosome evolution.
